# A Retrospective Review of Eye Complications in Patients with Ear, Nose, and Throat Disorders: Patterns of Recognition, Documentation, and Clinical Management

**DOI:** 10.7759/cureus.107076

**Published:** 2026-04-15

**Authors:** Saleh Khurshied, Hira G Shah, Ammara Aslam, Naeema Natasha, Mehrun Nisa, Muhammad A Zahid

**Affiliations:** 1 Otolaryngology - Head and Neck Surgery, Pakistan Institute of Medical Sciences, Islamabad, PAK; 2 Ophthalmology, Alshifa Trust Eye Hospital, Rawalpindi, PAK; 3 Otolaryngology - Head and Neck Surgery, Federal Government Polyclinic, Islamabad, PAK; 4 Medicine and Surgery, Pakistan Institute of Medical Sciences, Islamabad, PAK; 5 Ophthalmology, Monash Health, Clayton, AUS

**Keywords:** clincal audit, documentation errors, ent diseases, eye signs, hospital discharge, management rationale

## Abstract

Background

Ocular complications are known to be the result of various ENT conditions; the way they are reported and addressed in the discharge records is usually different. This retrospective study on discharge certificates aims to evaluate the trends in the identification of ENT disorders with ocular symptoms and signs, documentation quality, and clinical management done.

Methods

A total of 175 discharge certificates of patients who were admitted to the ENT department between February 2023 and February 2026 were audited retrospectively. Data gathered through patient demographics, initial ENT diagnosis, nature and degree of ocular complications, documentation completeness, treatment approaches, and follow-up recommendations. Clinically adapted criteria of the widely used classifications were used to categorize eye involvement as mild, moderate, or severe. The descriptive statistics were used to describe the trends and to compare the findings with the available literature.

Results

Out of 175, 113 (64.57%) were male patients, and 62 (35.43%) were female patients, and the mean age was 37 ± 11.37 years. The most common diagnosis was sinusitis with orbital extension at 84 (48%), and sinonasal tumors or nasopharyngeal tumors at 33 (18.86%) and 25 (14.29%), respectively. Mild cases of the eye involved the highest number of cases at 123 (70.29%), with moderate and severe cases taking 43 (24.57%) and 9 (5.14%), respectively. The rates of documentation were high concerning the identification of ocular findings in 166 (94.86%), the laterality in 170 (97.14%), and the follow-up or referral in 164 (93.71%). Severity grading was, however, recorded in only 38 (21.71%). Most of the management was conducted using medical therapy at 98 (56%), combined medical and surgery at 34 (19.43%), surgery alone at 17 (9.71%), and ophthalmology referral at 26 (14.9%).

Conclusions

Ocular complications in ENT disorders are relatively common but are not consistently detailed in discharge documentation, particularly with respect to severity. The use of structured discharge formats incorporating severity grading and interdisciplinary input may improve documentation consistency and clinical communication. These findings highlight potential areas for improvement in documentation practices and clinical standards; however, their impact on continuity of care and patient risk assessment was not directly evaluated in this study.

## Introduction

ENT-related ocular complications form a significant clinical problem, albeit with the tendency of being underemphasized in standard practice. Due to the anatomical proximity between ENT structures and the orbit, the pathological processes of infection, inflammation, trauma, or even surgical intervention can spread to the eye and influence the visual function [1,2].

It can be involved through direct disease transmission, inflammation, vascular or neural impairment, iatrogenic damage, or trauma. Certainly, these symptoms may be very diverse; a slight eyelid swelling, a reddening of the conjunctiva, or more severe results such as proptosis, loss of movement of the eyes, or blindness, depending on the pathology underlying the condition [2,3]. This must be identified early because late diagnoses can lead to permanent vision loss or systemic complications, particularly in susceptible populations like children. ENT-related ocular complications form a significant clinical problem, albeit with the tendency of being underemphasized in standard practice. Due to the anatomical proximity between ENT structures and the orbit, the pathological processes of infection, inflammation, trauma, or even surgical intervention can spread to the eye and influence the visual function [1,2]., elderly people, and immunocompromised individuals [3,4].

Discharge summaries are critical clinical and medico-legal records that describe the diagnoses, treatment administered, and patient outcome. They are also a useful tool in the retrospective audits conducted with the purpose of assessing the ways of recognizing, documenting, and treating ocular complications among ENT patients [5,6]. These types of analysis may reveal the lapses in paperwork, failures in following the guidelines, as well as general trends of clinical practice [5,7].

Available literature depicts that ocular complications depend on the age, ENT disease type, comorbidities, and even seasonal variations. Indicatively, orbital cellulitis and proptosis are more commonly registered in children and commonly exhibit seasonal accumulation, whereas odontogenic sinus involvement is more likely to display foreseeable patterns of eye involvement [1,2,6,8]. These trends are important to identify the risks to be stratified and diagnosed early, with multidisciplinary care being coordinated, as well as to help improve documentation practices [7,9].

Although there have been improvements in imaging, surgery, and shared care, ocular involvement is not well documented. A review of discharge certificates of various varieties of ENT conditions gives a wider picture of how these complications are treated in real-life situations. This research paper thus seeks to audit discharge summaries to identify common ENT disorders associated with ocular complications, describe their presentation, and evaluate existing documentation and management practices and gaps in our institutional setting.

## Materials and methods

Study design

This was a retrospective observational study.

Study objective

The primary objective of this study was to determine the frequency, pattern, and severity of ocular complications in patients admitted with ENT conditions, the quality of discharge certificates, and to evaluate associated demographic and clinical factors, and describe management strategies adopted for these complications.

Study setting

The study was conducted in the Department of Ear, Nose, and Throat - Head and Neck Surgery, Pakistan Institute of Medical Sciences (PIMS), Islamabad, Pakistan.

Study duration

The study was conducted over a 36-month period from February 2023 to February 2026.

Study population

Medical records (discharge cards) of patients admitted to the ENT (Otorhinolaryngology) department during the study period were reviewed.

Inclusion criteria

Patients of any age admitted with primary ENT conditions and having complete medical records were included. Complete records were defined as those containing detailed clinical notes, final diagnosis, treatment given, and documentation of ophthalmic findings where applicable.

Exclusion criteria

Patients with incomplete medical records, those with pre-existing ophthalmic conditions unrelated to the presenting ENT pathology, and patients who left against medical advice prior to complete evaluation were excluded from the study.

Data collection procedure

Data were collected using a predefined structured data collection proforma. The extracted variables included age, gender, primary ENT diagnosis, and presence and type of ocular complications. Ocular complications recorded included periorbital swelling, proptosis, eyelid erythema, conjunctival injection, ptosis, diplopia, restricted ocular movements, chemosis, periorbital ecchymosis, ocular pain, and visual disturbances. Data extraction was performed in a standardized manner, and records were reviewed systematically to ensure consistency. Documentation quality was assessed in terms of laterality, severity, and progression of ocular findings, where available. Management strategies were also recorded, including medical treatment, surgical intervention, and ophthalmology referral.

Classification of ocular complications

Ocular complications were categorized based on clinical severity as follows: (i) Mild: Periorbital edema, conjunctival injection, and minor visual complaints; (ii) Moderate: Proptosis, restricted ocular motility, and reduced visual acuity; (iii) Severe: Orbital abscess, cavernous sinus thrombosis, and permanent visual loss.

This classification was based on predefined clinical criteria in the literature [10].

Data analysis

Data were entered and analyzed using Microsoft Excel (Microsoft Corp., Redmond, WA, USA) and IBM SPSS Statistics for Windows, version 26 (IBM Corp., Armonk, NY, USA). Descriptive statistics were used to summarize the data. Categorical variables were presented as frequencies and percentages, while continuous variables were expressed as mean ± standard deviation.

Ethical considerations

Approval for the study was obtained from the Head of the Department prior to commencement. Patient confidentiality was strictly maintained, and all identifying information was removed during data collection and analysis. The institutional ethics committee waived the requirement for informed consent due to the retrospective nature of the study.

## Results

A total of 175 discharge certificates of patients with ENT-related ocular complications were compared. Out of them, 113 patients (64.57%) were male patients and 62 (35.43%) female patients. The average age was 37 ± 11.37 years between the age range of 4-70 years.

Table 1 shows the primary ENT diagnosis distributions. The most common one was sinusitis (acute or chronic) with orbital extension.

**Table 1:** Distribution of ENT disorders Total number of cases (N): 175 (100%). ENT: ear, nose, and throat

ENT disorder	Number of patients (N)	Percentage (%)
Sinusitis (acute/chronic) with orbital extension	84	48
Sinonasal or nasopharyngeal tumors affecting orbit	33	18.86
Mucormycosis or invasive fungal sinusitis	25	14.29
Facial trauma involving orbital or periorbital region	21	12
Thyroid-related eye disease	12	6.86
Total	175	100

**Figure 1:**
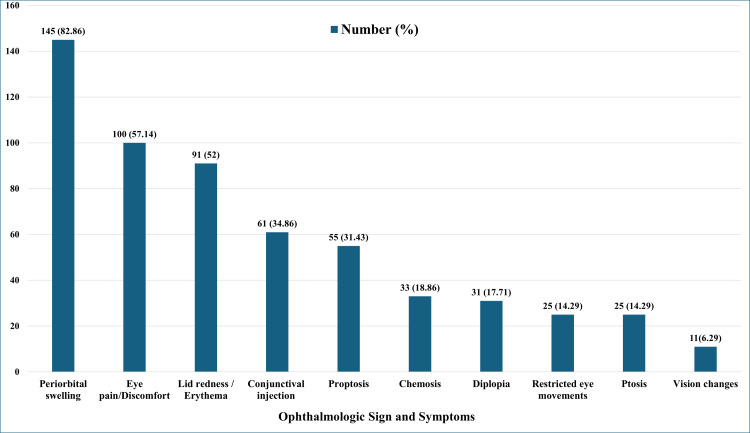
Distribution of documented ophthalmological signs and symptoms y-axis (number of cases): The number of certificates with specific ophthalmological signs and symptoms. x-axis (ophthalmological signs and symptoms): The different ophthalmological signs and symptoms according to the checklist.

The ophthalmologic signs and symptoms are recorded, as below (Figure 1). The most common is periorbital swelling, followed by eye pain, lid redness, and as shown in Figure 1.

Table 2 indicates the severity distribution of ocular complications. The most prevalent were mild, moderate, and severe involvement.

**Table 2:** Classification of severity of eye complications Total number of cases (N): 175 (100%). Adopted from existing guidelines [10].

Severity	Clinical features	Number of patients	Percentage (%)
Mild	Periorbital edema, conjunctival injection, minor visual complaints	123	70.29
Moderate	Proptosis, restricted ocular motility, reduced visual acuity	43	24.57
Severe	Orbital abscess, cavernous sinus thrombosis, permanent visual loss	9	5.14

Documentation quality (Table 3) assessment revealed high ocular findings recording, laterality recording, and follow-up recommendation rates. On the contrary, classification into severity was observed in a significantly lower number of cases.

**Table 3:** Completeness of different documentation parameters Total number of cases (N): 175 (100%).

Documentation parameter	Documented	Not documented	Percentage documented (%)
Presence of ocular findings	166	9	94.86
Laterality of eye involvement	170	5	97.14
Severity classification	38	147	21.71
Follow-up/ophthalmology referral	164	11	93.71
Procedures/interventions/management done during admission	101	74	57.7
Investigations/imaging	155	20	88.57

The management approaches (Table 4) were different based on clinical presentation and severity. A majority of the patients were treated using medical therapy only, but some needed to use a combination or surgery.

**Table 4:** Management of eye complications Total number of cases (N): 175 (100%).

Management Strategy	Number of Patients	Percentage (%)
Medical therapy only (Topical or systemic)	98	56
Combined medical + surgical approach	34	19.43
Ophthalmology referral	26	14.86
Surgical intervention (e.g., drainage)	17	9.71

## Discussion

Sinusitis with orbital extension was the primary diagnosis that accompanied ocular complications in the most prevalent cases in this retrospective review of 175 discharge summaries, 84 (48%). This was then followed by sinonasal or nasopharyngeal tumors at 33 (18.9%), invasive fungal sinusitis, which also included mucormycosis, at 25 (14.3%). These are in line with other literature that have been published in the past, and sinus-related disease is widely cited as the primary cause of orbital and periocular complications in ENT practice [1]. Indicatively, the article by Fernandes et al. noted that sinusitis is a causative agent of almost 79% of cases of orbital complication, advocating its clinical implications [11].

In terms of the severity of patients in the present study, most of them presented with mild cases of ocular complications 123 (70.3%). This trend can be compared to other reports in the literature. Cases with early-stage orbital cellulitis and periorbital inflammation were reported by Tsirouki et al. to constitute about 62% of their cases and normally showed edema and conjunctival changes [12]. In our group, moderate complications were noted in 43 (24.6%) of the patients and this is quite consistent with the results provided by Alabbasi et al., who noted moderate complication in 23.5% of similar cases [4]. The number of severe complications was not very high at 9 (5.1%), which is also similar to the findings of Mejia et al., where only 6.4% of cases were proven to progress to further conditions of orbital abscess or cavernous sinus thrombosis [6]. These extreme manifestations are clinically significant although less common because of the possibility of morbidity.

The documentation practices analysis showed that there was a high level of compliance in some major areas. There were records of ocular findings in 166 (94.9%) discharge summaries, laterality 170 (97.1%) and follow-up instructions in 164 (93.7%). Though these figures are a good practice in general, they do indicate that some areas can be improved. Similar investigations have indicated greater variability; for example, Gobind & Sachdeva have reported ocular findings in 78% and laterality in 82% [5].

One of the gaps that were observed during this study was that the severity classification was low in only 38 (21.7%) discharge certificates. This absence of documented severity could hinder efficient communication at clinical handover and minimize the capacity to rank patient risk. There are existing systems, including Chandler classification, which might be used to standardize this element of reporting [10].

In the context of investigations, the imaging was reported in 88.6% and this is in tandem with the present clinical practice. CT and MRI modalities are regularly used to evaluate the issue of orbital involvement especially where there is a suspicion of sinus disease or an indication of complications. In like cases of clinical scenarios, similar audits have also documented imaging documentation rates of 85-90% [13].

Conversely, the procedure/intervention documentation was less steady since it was found in only 74 (57.7%) of discharge summaries. It is a bit lower than the rates which have been reported in other studies, the rates being observed to range between 68% and 74% [5]. Missing documentation of interventions would impact continuity of care, particularly among patients who need aftercare or multidisciplinary care.

The patterns of management that were witnessed in this research are mostly the clinical practices that are in existence. The most frequent intervention was medical therapy, practiced in 98 (56%) of the patients. This observation concurs with the available evidence that most (about 60-65) cases of early or uncomplicated orbital cases do well with systemic antibiotics and supportive care [11,12,14]. In 34 (19.4%), combined medical and surgical treatment was used and isolated surgical intervention was necessary in 17 (9.7%). These percentages are comparable with those in other studies, with combined methods having around 20-22% and primary surgical treatment 8-12% of cases [6,11].

The Ophthalmology referral was recorded in 26 (14.9%) of patients. This figure can be considered rather low; however, it is not out of the scope of those found in multidisciplinary environments, where the rate of referral ranges between 12% and 28% based on the practice of the institutional setting and severity of a case [5,13]. Such variation underscores variation in referral levels, as well as interdisciplinary collaboration levels, among centers.

Collectively, the above findings demonstrate the need to adopt a more formalized process of reporting ocular involvement in ENT patients. Specifically, the regular application of severity grading systems, including Chandler classification, may enhance the level of clarity, interspecialty communication, and help undertake risk assessment more accurately [10]. Unified discharge templates can also contribute to the quality of documentation.

Generally, this audit supports the idea that ocular complication of ENT disorders is quite prevalent yet not always well-presented in the discharge documentation, particularly when it comes to the extent of its severity. These gaps may be resolved by designing documentation practices and enhancing the interdisciplinary coordination of care and clinical outcomes [14,15].

A few shortcomings of this study should be noted. The retrospective type of design implies that the results lack the accuracy and completeness of the clinical evaluation but rely on the completeness and accuracy of the available records. Also, this was a study that was done in one center and thus could not be generalized. There is also a limitation on assessment of post-discharge patient outcomes due to the lack of long-term follow-up data. In spite of these limitations, the study can be useful in understanding the current practices and where they can be enhanced.

## Conclusions

The most prevalent ENT condition associated with ocular complications in this cohort was sinusitis with orbital extension. Although the majority of cases were mild (123; 70.28%), the presence of moderate (43; 24.57%) and severe (9; 5.14%) involvement highlights the importance of early recognition. Periorbital swelling, eye discomfort, and eye redness were among the most frequently noted ocular findings. Documentation was generally high for ocular findings, laterality, and follow-up recommendations; however, severity grading was recorded in only 38 (21.71%) cases, indicating a notable gap in documentation practices. Management was predominantly medical (98; 56%), with combined or surgical approaches used in more severe cases, and ophthalmology referrals documented in 26 (14.86%) patients.

The findings show common ENT disorders presenting as ocular symptoms, variability in documentation, and management practices. Further work may focus on standardizing discharge documentation using structured templates to improve completeness and consistency.
